# 2-Methyl-*N*-*p*-tolyl­benzamide: a second monoclinic polymorph

**DOI:** 10.1107/S1600536810010378

**Published:** 2010-03-24

**Authors:** Aamer Saeed, Rasheed Ahmad Khera, Jim Simpson

**Affiliations:** a Department of Chemistry, Quaid-i-Azam University, Islamabad 45320, Pakistan; b Department of Chemistry, University of Otago, PO Box 56, Dunedin, New Zealand

## Abstract

The title compound, C_15_H_15_NO, (I), is a polymorph of the structure (II) reported by Gowda *et al.* [*Acta Cryst.* (2008), E**64**, o1494]. Compound (II) crystalllizes in the space group *C*2/*c* (Z = 8), whereas the title compound occurs in space group *P*2_1_/*c* (*Z* = 4). The two mol­ecular structures differ slightly in the relative orientations of their central amide group with respect to the benzoyl ring [dihedral angles of 55.99 (7) for (I) and 59.96 (11)° for (II)] and in the inclination of the benzoyl and aniline rings [88.67 (8) for (I) and 81.44 (5)° for (II)]. In the crystal structure of (I), mol­ecules are linked by N—H⋯O hydrogen bonds, forming *C*(4) chains, which are augmented by weak C—H⋯O inter­actions. The structure is further stabilized by C—H⋯π contacts involving both of the aromatic rings.

## Related literature

For the biological activity of *N*-substituted benzamides, see: Olsson *et al.* (2002 ▶); Lindgren *et al.* (2001 ▶). For the use of heterocyclic analogs of benzanilide derivatives as potassium channel activators, see: Calderone *et al.* (2006 ▶). For the use of 2-nitro­benzamides in organic synthesis, see: Zhichkin *et al.* (2007 ▶); Beccalli *et al.* (2005 ▶). For the original monoclinic polymorph, see: Gowda *et al.* (2008 ▶). For the related *N*-(2,4-dimethyl­phen­yl)-2-methyl­benzamide, see: Gowda *et al.* (2009 ▶). For hydrogen-bond motifs, see: Bernstein *et al.* (1995 ▶).
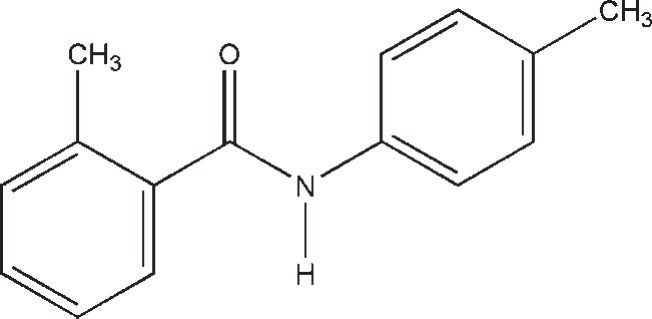


## Experimental

### 

#### Crystal data

C_15_H_15_NO
*M* *_r_* = 225.28
Monoclinic, 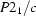

*a* = 20.259 (3) Å
*b* = 7.0681 (10) Å
*c* = 8.7941 (13) Å
β = 95.942 (9)°
*V* = 1252.5 (3) Å^3^
*Z* = 4
Mo *K*α radiation
μ = 0.08 mm^−1^
*T* = 89 K
0.30 × 0.19 × 0.06 mm

#### Data collection

Bruker APEXII CCD diffractometer
Absorption correction: multi-scan (*SADABS*; Bruker, 2006 ▶) *T*_min_ = 0.803, *T*_max_ = 1.000
8447 measured reflections
1283 independent reflections
1028 reflections with *I* > 2σ(*I*)
*R*_int_ = 0.052
θ_max_ = 20.7°

#### Refinement

*R*[*F* ^2^ > 2σ(*F* ^2^)] = 0.040
*wR*(*F* ^2^) = 0.108
*S* = 1.07
1283 reflections
159 parameters
H atoms treated by a mixture of independent and constrained refinement
Δρ_max_ = 0.16 e Å^−3^
Δρ_min_ = −0.23 e Å^−3^

### 

Data collection: *APEX2* (Bruker, 2006 ▶); cell refinement: *APEX2* and *SAINT* (Bruker, 2006 ▶); data reduction: *SAINT*; program(s) used to solve structure: *SHELXS97* (Sheldrick, 2008 ▶); program(s) used to refine structure: *SHELXL97* (Sheldrick, 2008 ▶) and *TITAN2000* (Hunter & Simpson, 1999 ▶); molecular graphics: *SHELXTL* (Sheldrick, 2008 ▶) and Mercury (Macrae *et al.*, 2008 ▶); software used to prepare material for publication: *SHELXL97*, *enCIFer* (Allen *et al.*, 2004 ▶), *PLATON* (Spek, 2009 ▶) and *publCIF* (Westrip, 2010 ▶).

## Supplementary Material

Crystal structure: contains datablocks global, I. DOI: 10.1107/S1600536810010378/hb5364sup1.cif

Structure factors: contains datablocks I. DOI: 10.1107/S1600536810010378/hb5364Isup2.hkl

Additional supplementary materials:  crystallographic information; 3D view; checkCIF report

## Figures and Tables

**Table 1:** Hydrogen-bond geometry (Å, °) *Cg*1 and *Cg*2 are the centroids of the C3–C7 and C8–C13 benzene rings, repectively.

*D*—H⋯*A*	*D*—H	H⋯*A*	*D*⋯*A*	*D*—H⋯*A*
N1—H1*N*⋯O1^i^	0.90 (3)	1.94 (3)	2.821 (3)	169 (2)
C9—H9⋯O1^i^	0.95	2.71	3.366 (3)	127
C7—H7⋯*Cg*2^ii^	0.95	2.84	3.751 (3)	160
C31—H31*C*⋯*Cg*1^iii^	0.98	2.86	3.676 (3)	141

Symmetry codes: (i) 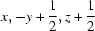; (ii) 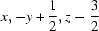; (iii) 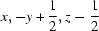.

**Fig. 1.**
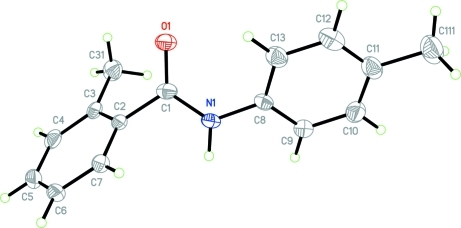
The structure of (I) with displacement ellipsoids for the non-hydrogen atoms drawn at the 50% probability level.

**Fig. 2.**
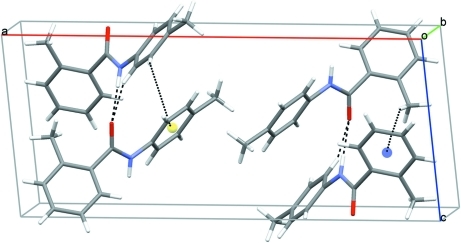
Crystal packing for (I) viewed down the b axis with hydrogen bonds drawn as dashed lines and C—H···π interactions drawn as dotted lines. The blue and yellow spheres represent centroids of the benzene rings.

## supplementary crystallographic information

### Comment

N-substituted benzamides are well known anticancer compounds and the mechanism of action for N-substituted benzamide-induced apoptosis has been studied, using declopramide as a lead compound (Olsson *et al.*, 2002). N-substituted benzamides inhibit the activity of nuclear factor- B and nuclear factor T cell activity while inducing activator protein 1 activity in T lymphocytes (Lindgren *et al.*, 2001). Heterocyclic analogs of benzanilide derivatives are potassium channel activators (Calderone *et al.*, 2006). *N*-alkylated 2-nitrobenzamides are intermediates in the synthesis of dibenzo[b,e][1,4]diazepines (Zhichkin *et al.*, 2007) and *N*-acyl-2-nitrobenzamides are precursors of 2,3-disubstitued 3*H*-quinazoline-4-ones (Beccalli *et al.*, 2005).

The title compound, (I), is a second monoclinic polymorph of the structure of this benzamide derivative which crystallises in the space group P21/c. An alternative structure, II, in the space group C2/c was reported previously by Gowda *et al.*, (2008). The major structural differences between the two polymorphs lie in the orientations of their central C2,C1,O1,N1,C8 amide groups with respect to the C2···C6 benzoyl ring. In I the N1–C1–C2–C7 dihedral angle is 55.69 (3) for (I) whereas for (II) it is -60.69 (18). Furthermore the angle between the plane through C2,C1,O1,N1,C8 and the C2···C6 ring plane is 55.99 (7)/% in (I) but 59.96 (11) for (II) and the two phenyl rings are respectively inclined at 88.67 (8)/% for (I) and 81.44 (5)/% for (II). Bond distances in the molecule are normal and comparable to those in the second polymorph and in a closely related benzamide derivative (Gowda *et al.*, 2009).

In the crystal structure intermolecular N1–H1···O1 hydrogen bonds form C(4) chains down the *c* axis (Bernstein *et al.* 1995). These chains are further stabilised by weak C9–H9···O1 interactions and C–H···π contacts involving both the aniline and benzoyl ring systems.

### Experimental

2-Methylbenzoyl chloride (1 mmol) in CHCl_3_ was treated with 4-methylaniline (3.5 mmol) under a nitrogen atmosphere at reflux for 3.5 h. Upon cooling, the reaction mixture was diluted with CHCl_3_ and washed consecutively with 1 *M* aq HCl and saturated aq NaHCO_3_. The organic layer was dried over anhydrous sodium sulfate and concentrated under reduced pressure. Crystallization of the residue from ethanol afforded colourless plates of (I) in 78% yield: Anal. calcd. for C_15_H_25_N_O_: C, 79.97; H, 6.71; N, 6.22; found: C, 80.06; H, 6.87; N, 6.01%

### Refinement

The H atom bound to N1 was located in a difference map and refined isotropically. All other H-atoms were positioned geometrically and refined using a riding model with d(C—H) = 0.95 Å, U_iso_ = 1.2U_eq_ (C) for aromatic and d(C—H) = 0.98 Å, U_iso_ = 1.5U_eq_ (C) for methyl C atoms. Crystals were very thin and weakly diffracting. Even with 60 s scans over 24 h, no useful data was observed beyond theta = 20.71 °.

### Crystal data

**Table:**

C_15_H_15_NO	*F*(000) = 480
*M**_r_* = 225.28	*D*_x_ = 1.195 Mg m^−^^3^
Monoclinic, *P*2_1_/*c*	Mo *K*α radiation, λ = 0.71073 Å
Hall symbol: -P 2ybc	Cell parameters from 1469 reflections
*a* = 20.259 (3) Å	θ = 3.1–20.2°
*b* = 7.0681 (10) Å	µ = 0.08 mm^−^^1^
*c* = 8.7941 (13) Å	*T* = 89 K
β = 95.942 (9)°	Rectangular plate, colourless
*V* = 1252.5 (3) Å^3^	0.30 × 0.19 × 0.06 mm
*Z* = 4	

### Data collection

**Table:**

Bruker APEXII CCD diffractometer	1283 independent reflections
Radiation source: fine-focus sealed tube	1028 reflections with *I* > 2σ(*I*)
graphite	*R*_int_ = 0.052
ω scans	θ_max_ = 20.7°, θ_min_ = 1.0°
Absorption correction: multi-scan (*SADABS*; Bruker, 2006)	*h* = −20→20
*T*_min_ = 0.803, *T*_max_ = 1.000	*k* = −7→7
8447 measured reflections	*l* = −8→8

### Refinement

**Table:**

Refinement on *F*^2^	Primary atom site location: structure-invariant direct methods
Least-squares matrix: full	Secondary atom site location: difference Fourier map
*R*[*F*^2^ > 2σ(*F*^2^)] = 0.040	Hydrogen site location: inferred from neighbouring sites
*wR*(*F*^2^) = 0.108	H atoms treated by a mixture of independent and constrained refinement
*S* = 1.07	*w* = 1/[σ^2^(*F*_o_^2^) + (0.0571*P*)^2^ + 0.3937*P*] where *P* = (*F*_o_^2^ + 2*F*_c_^2^)/3
1283 reflections	(Δ/σ)_max_ = 0.002
159 parameters	Δρ_max_ = 0.16 e Å^−^^3^
0 restraints	Δρ_min_ = −0.23 e Å^−^^3^

### Special details

**Table:**

Geometry. All esds (except the esd in the dihedral angle between two l.s. planes) are estimated using the full covariance matrix. The cell esds are taken into account individually in the estimation of esds in distances, angles and torsion angles; correlations between esds in cell parameters are only used when they are defined by crystal symmetry. An approximate (isotropic) treatment of cell esds is used for estimating esds involving l.s. planes.
Refinement. Refinement of *F*^2^ against ALL reflections. The weighted *R*-factor wR and goodness of fit *S* are based on *F*^2^, conventional *R*-factors *R* are based on *F*, with *F* set to zero for negative *F*^2^. The threshold expression of *F*^2^ > σ(*F*^2^) is used only for calculating *R*-factors(gt) etc. and is not relevant to the choice of reflections for refinement. *R*-factors based on *F*^2^ are statistically about twice as large as those based on *F*, and *R*- factors based on ALL data will be even larger.

### Fractional atomic coordinates and isotropic or equivalent isotropic displacement parameters (Å^2^)

**Table:**

	*x*	*y*	*z*	*U*_iso_*/*U*_eq_	
N1	0.73575 (10)	0.2686 (3)	0.6898 (2)	0.0250 (6)	
H1N	0.7455 (11)	0.297 (3)	0.789 (3)	0.030*	
O1	0.76424 (8)	0.0912 (2)	0.48978 (19)	0.0301 (5)	
C1	0.77164 (12)	0.1340 (3)	0.6268 (3)	0.0231 (7)	
C2	0.82221 (12)	0.0392 (3)	0.7367 (3)	0.0207 (7)	
C3	0.88871 (13)	0.0342 (3)	0.7074 (3)	0.0232 (7)	
C31	0.91234 (13)	0.1250 (4)	0.5675 (3)	0.0327 (7)	
H31A	0.8943	0.0557	0.4760	0.049*	
H31B	0.9609	0.1217	0.5753	0.049*	
H31C	0.8972	0.2567	0.5602	0.049*	
C4	0.93384 (13)	−0.0562 (3)	0.8148 (3)	0.0269 (7)	
H4	0.9795	−0.0597	0.7988	0.032*	
C5	0.91342 (14)	−0.1405 (3)	0.9437 (3)	0.0302 (7)	
H5	0.9450	−0.2013	1.0149	0.036*	
C6	0.84744 (13)	−0.1368 (3)	0.9697 (3)	0.0285 (7)	
H6	0.8334	−0.1961	1.0579	0.034*	
C7	0.80182 (12)	−0.0460 (3)	0.8663 (3)	0.0239 (7)	
H7	0.7564	−0.0419	0.8840	0.029*	
C8	0.68730 (12)	0.3892 (4)	0.6124 (3)	0.0230 (7)	
C9	0.68482 (12)	0.5755 (4)	0.6619 (3)	0.0278 (7)	
H9	0.7146	0.6179	0.7455	0.033*	
C10	0.63886 (13)	0.6993 (4)	0.5893 (3)	0.0330 (7)	
H10	0.6381	0.8270	0.6228	0.040*	
C11	0.59398 (13)	0.6409 (4)	0.4687 (3)	0.0327 (8)	
C111	0.54250 (14)	0.7756 (4)	0.3928 (3)	0.0466 (9)	
H11A	0.5237	0.7223	0.2949	0.070*	
H11B	0.5634	0.8975	0.3751	0.070*	
H11C	0.5071	0.7939	0.4594	0.070*	
C12	0.59739 (13)	0.4542 (4)	0.4208 (3)	0.0345 (8)	
H12	0.5677	0.4116	0.3370	0.041*	
C13	0.64310 (12)	0.3286 (4)	0.4923 (3)	0.0285 (7)	
H13	0.6440	0.2009	0.4588	0.034*	

### Atomic displacement parameters (Å^2^)

**Table:**

	*U*^11^	*U*^22^	*U*^33^	*U*^12^	*U*^13^	*U*^23^
N1	0.0360 (14)	0.0248 (14)	0.0135 (12)	0.0036 (11)	−0.0006 (11)	−0.0010 (11)
O1	0.0457 (12)	0.0259 (11)	0.0182 (12)	0.0017 (9)	0.0006 (9)	−0.0010 (8)
C1	0.0349 (17)	0.0173 (16)	0.0170 (17)	−0.0079 (13)	0.0029 (13)	−0.0008 (12)
C2	0.0319 (17)	0.0133 (14)	0.0163 (15)	0.0013 (12)	−0.0004 (13)	−0.0022 (12)
C3	0.0354 (18)	0.0108 (14)	0.0232 (16)	0.0002 (12)	0.0020 (13)	−0.0042 (12)
C31	0.0396 (17)	0.0253 (17)	0.0341 (17)	−0.0008 (13)	0.0079 (13)	0.0024 (13)
C4	0.0317 (16)	0.0177 (16)	0.0309 (18)	0.0009 (12)	0.0015 (13)	−0.0073 (13)
C5	0.042 (2)	0.0206 (16)	0.0259 (18)	0.0066 (13)	−0.0043 (14)	−0.0010 (13)
C6	0.046 (2)	0.0204 (16)	0.0196 (16)	0.0028 (13)	0.0045 (14)	0.0034 (12)
C7	0.0310 (16)	0.0193 (15)	0.0214 (16)	0.0020 (12)	0.0018 (13)	−0.0006 (12)
C8	0.0278 (15)	0.0261 (17)	0.0154 (15)	0.0022 (13)	0.0034 (13)	0.0045 (13)
C9	0.0325 (16)	0.0289 (18)	0.0217 (16)	0.0044 (13)	0.0020 (13)	−0.0007 (13)
C10	0.0418 (18)	0.0284 (17)	0.0299 (17)	0.0083 (14)	0.0088 (15)	0.0023 (14)
C11	0.0317 (17)	0.040 (2)	0.0267 (17)	0.0069 (14)	0.0062 (14)	0.0112 (14)
C111	0.0442 (19)	0.054 (2)	0.0430 (19)	0.0141 (16)	0.0079 (15)	0.0182 (16)
C12	0.0324 (17)	0.043 (2)	0.0271 (17)	−0.0043 (14)	−0.0004 (13)	0.0054 (14)
C13	0.0330 (16)	0.0273 (17)	0.0252 (17)	−0.0013 (14)	0.0020 (14)	0.0045 (13)

### Geometric parameters (Å, °)

**Table:**

N1—C1	1.351 (3)	C6—H6	0.9500
N1—C8	1.420 (3)	C7—H7	0.9500
N1—H1N	0.90 (3)	C8—C13	1.380 (3)
O1—C1	1.236 (3)	C8—C9	1.389 (3)
C1—C2	1.493 (3)	C9—C10	1.385 (3)
C2—C7	1.389 (3)	C9—H9	0.9500
C2—C3	1.398 (3)	C10—C11	1.387 (4)
C3—C4	1.399 (3)	C10—H10	0.9500
C3—C31	1.509 (4)	C11—C12	1.389 (4)
C31—H31A	0.9800	C11—C111	1.515 (4)
C31—H31B	0.9800	C111—H11A	0.9800
C31—H31C	0.9800	C111—H11B	0.9800
C4—C5	1.381 (4)	C111—H11C	0.9800
C4—H4	0.9500	C12—C13	1.386 (4)
C5—C6	1.380 (4)	C12—H12	0.9500
C5—H5	0.9500	C13—H13	0.9500
C6—C7	1.386 (3)		
			
C1—N1—C8	126.9 (2)	C6—C7—C2	120.3 (2)
C1—N1—H1N	118.9 (16)	C6—C7—H7	119.8
C8—N1—H1N	113.8 (16)	C2—C7—H7	119.8
O1—C1—N1	123.8 (2)	C13—C8—C9	119.5 (2)
O1—C1—C2	121.8 (2)	C13—C8—N1	122.8 (2)
N1—C1—C2	114.4 (2)	C9—C8—N1	117.7 (2)
C7—C2—C3	121.0 (2)	C10—C9—C8	119.9 (2)
C7—C2—C1	118.9 (2)	C10—C9—H9	120.0
C3—C2—C1	120.1 (2)	C8—C9—H9	120.0
C2—C3—C4	117.5 (2)	C9—C10—C11	121.4 (3)
C2—C3—C31	122.2 (2)	C9—C10—H10	119.3
C4—C3—C31	120.3 (2)	C11—C10—H10	119.3
C3—C31—H31A	109.5	C10—C11—C12	117.8 (2)
C3—C31—H31B	109.5	C10—C11—C111	121.1 (3)
H31A—C31—H31B	109.5	C12—C11—C111	121.1 (3)
C3—C31—H31C	109.5	C11—C111—H11A	109.5
H31A—C31—H31C	109.5	C11—C111—H11B	109.5
H31B—C31—H31C	109.5	H11A—C111—H11B	109.5
C5—C4—C3	121.4 (2)	C11—C111—H11C	109.5
C5—C4—H4	119.3	H11A—C111—H11C	109.5
C3—C4—H4	119.3	H11B—C111—H11C	109.5
C6—C5—C4	120.4 (2)	C13—C12—C11	121.5 (2)
C6—C5—H5	119.8	C13—C12—H12	119.2
C4—C5—H5	119.8	C11—C12—H12	119.2
C5—C6—C7	119.4 (2)	C8—C13—C12	119.9 (3)
C5—C6—H6	120.3	C8—C13—H13	120.0
C7—C6—H6	120.3	C12—C13—H13	120.0
			
C8—N1—C1—O1	−3.8 (4)	C3—C2—C7—C6	0.4 (3)
C8—N1—C1—C2	175.9 (2)	C1—C2—C7—C6	179.3 (2)
O1—C1—C2—C7	−124.6 (3)	C1—N1—C8—C13	38.7 (4)
N1—C1—C2—C7	55.7 (3)	C1—N1—C8—C9	−141.7 (2)
O1—C1—C2—C3	54.3 (3)	C13—C8—C9—C10	−0.9 (4)
N1—C1—C2—C3	−125.4 (2)	N1—C8—C9—C10	179.4 (2)
C7—C2—C3—C4	−1.3 (3)	C8—C9—C10—C11	1.1 (4)
C1—C2—C3—C4	179.8 (2)	C9—C10—C11—C12	−1.2 (4)
C7—C2—C3—C31	179.9 (2)	C9—C10—C11—C111	178.2 (2)
C1—C2—C3—C31	1.0 (3)	C10—C11—C12—C13	1.2 (4)
C2—C3—C4—C5	1.1 (3)	C111—C11—C12—C13	−178.1 (2)
C31—C3—C4—C5	−180.0 (2)	C9—C8—C13—C12	1.0 (4)
C3—C4—C5—C6	−0.2 (4)	N1—C8—C13—C12	−179.4 (2)
C4—C5—C6—C7	−0.7 (4)	C11—C12—C13—C8	−1.1 (4)
C5—C6—C7—C2	0.6 (4)		

### Hydrogen-bond geometry (Å, °)

**Table:**

Cg1 and Cg2 are the centroids of the C3–C7 and C8–C13 benzene rings, repectively.

**Table:**

*D*—H···*A*	*D*—H	H···*A*	*D*···*A*	*D*—H···*A*
N1—H1N···O1^i^	0.90 (3)	1.94 (3)	2.821 (3)	169 (2)
C9—H9···O1^i^	0.95	2.71	3.366 (3)	127
C7—H7···Cg2^ii^	0.95	2.84	3.751 (3)	160
C31—H31C···Cg1^iii^	0.98	2.86	3.676 (3)	141

Symmetry codes: (i) *x*, −*y*+1/2, *z*+1/2; (ii) *x*, −*y*+1/2, *z*−3/2; (iii) *x*, −*y*+1/2, *z*−1/2.
